# An Assessment of Harm in Adults—Adverse Childhood Experiences Screening in Primary Care: A Survey-Based Study

**DOI:** 10.1177/23743735251344505

**Published:** 2025-06-05

**Authors:** Katelyn M. Inch, Craig Olmstead, Brenna A. Kaschor

**Affiliations:** 1 Department of Health Sciences, 6221 Western University, London, Ontario, Canada; 2 Prisma Health Care Collaborative, London, Ontario, Canada; 3 Department of Family Medicine, 70384 Schulich School of Medicine and Dentistry, 6221 Western University, London, Ontario, Canada

**Keywords:** Adverse childhood experience, family practice/primary care, clinical practice guidelines, patient experiences

## Abstract

The Adverse Childhood Experiences Questionnaire (ACE-Q) screens for adverse childhood experiences (ACEs), which are linked to increased disease risk. Although pediatric studies report no adverse effects of ACE-Q use, primary care data is limited. This study examined adult patients’ experiences with ACE-Q screening in primary care. Adults (18+) at a primary care center in London, Ontario, completed the ACE-Q and a follow-up questionnaire evaluating ACE screening experience. Correlations assessed relationships between ACE-Q scores and follow-up responses. Among 260 participants, 81% reported at least one ACE. Most (82%) felt comfortable discussing stressful childhood experiences with their healthcare provider. Higher ACE scores were associated with increased discomfort (r_s_ = −0.166, *P* = 0.007), feeling upset by the ACE-Q (r_s_ = 0.173, *P* = 0.005), and greater interest in learning about ACEs (r_s_ = 0.177, *P* = 0.004). Overall, ACE-Q screening in primary care was generally well-received, with most patients recognizing its relevance despite some discomfort. These findings highlight the potential for integrating ACE screening into routine primary care to address long-term health risks. Further research is needed to confirm findings and optimize screening practices.

## Introduction

The Adverse Childhood Experiences Questionnaire (ACE-Q) screens for 10 categories of traumatic or stressful life events occurring before age 18.^1^ These events fall into three domains: abuse (physical, emotional, sexual), neglect (physical, emotional), and household dysfunction (substance use, mental illness, parental separation, domestic violence, or incarceration of a family member). Each event contributes one point to the total ACE-Q score, with higher scores strongly linked to an increased risk of disease later in life.^2^

Adverse childhood experiences (ACEs) influence long-term health through biological and behavioral pathways, including chronic stress activation and maladaptive coping mechanisms.^3^ Persistent stress responses, especially in the absence of supportive relationships or stable environments, can lead to long-term physiological and psychological effects. Over time, individuals may develop heightened survival mechanisms that, while protective in childhood, may contribute to difficulties in emotional regulation, relationships, and increased vulnerability to mental and physical health challenges in adulthood.^4–6^

Despite the well-documented health risks associated with ACEs, these experiences are often overlooked in routine primary care assessments,^7,8^ even though prevention is a key focus of primary care. One concern regarding ACE screening is the potential for re-traumatization or distress.^9^ However, pediatric studies indicate that screening does not negatively impact therapeutic relationships or cause significant distress,^6,10,11^ though research on ACE screening in adult primary care populations remains limited. The objective of this study was to assess the experience of ACE screening for adult patients in a primary care setting.

## Methodology

The ACE-Q is a validated tool promoted for patient use by the CDC and WHO^12,13^ and is reliable as a retrospective assessment of ACEs.^14^ It is important to note that in our version of the ACE-Q, based on the well-established version of the ACE-Q used by the Center for Youth Wellness (CYW ACE-Q), individuals provide the total number of ACEs, but do not indicate which events were experienced (Appendix 1).^15^ Based on concerns raised around the use of ACE screening in adult primary care patients, our team developed a brief follow-up questionnaire aimed at understanding the patient's experience of completing the ACE-Q.

### Inclusion Criteria

Participants were eligible if they were 18 years or older, registered patients at a primary care center in London, Ontario, had an email on file, and had previously agreed to receive research invitations.

### Exclusion Criteria

Patients were excluded if they were under 18 years of age or had opted out of research participation.

### Participant Recruitment

Eligible participants were contacted through email via a secure patient messaging portal (Ocean – Cognisant MD) between May 2021 and November 2022. Email addresses were previously collected by physicians during patient rostering, and those contacted had consented to receive invitations for relevant research initiatives. This study adhered to ethical guidelines for human subjects research and received approval from an independent institutional review board. No ethical issues arose during the research process, as participation was voluntary, responses were anonymous, and no identifiable personal information was collected. Electronic informed consent to participate was obtained before participation. The use of the remote data collection feature created an opportunity for the patient to participate in a low-pressure environment.

After providing informed consent, patients were directed to complete the ACE-Q electronically via the same secure survey platform. The ACE-Q included a brief introductory paragraph explaining the concept of ACEs and their potential impact on health. Providing this introductory paragraph ensured that participants had a basic understanding of the purpose of ACE screening, which is an important component of informed consent. Following completion of the ACE-Q, participants were immediately presented with a short follow-up questionnaire assessing their experience with ACE screening. The follow-up questionnaire asked about comfort level, preferred form of questionnaire administration, comprehension of the importance of ACE screening, feelings of re-traumatization, and opportunities for more information, using a 7-point Likert scale ranging from “Strongly Disagree” to “Strongly Agree.” The questionnaire also included general demographic information (Appendix 2). The follow-up questionnaire was devised by our team to allow for deeper understanding of patients’ experiences and feelings after completing ACE screening. Items were selected based on the team's clinical expertise and knowledge of key considerations in ACE screening, such as patient comfort and understanding. While we did not formally pilot-test the questionnaire or conduct a literature review specific to questionnaire development, the questions were reviewed collaboratively by team members to ensure they were clear, concise, and relevant to the study objectives. This pragmatic approach allowed us to gather initial insights into patient perspectives on ACE screening in a primary care setting.

### Statistical Analysis

Analyses of data were performed using IBM SPSS Statistics.^16^ ACE scores were analyzed as a continuous and discrete variable to provide a comprehensive understanding of how ACEs may relate to questionnaire responses. As a continuous variable, analysis was done using scores of 0-10, with an equal difference between each integer. As a discrete variable, ACE scores were split into clinically relevant categories of low (ACE score 0-3), high (4-6), and very high (7-10) based on established thresholds in the literature.^3^ This categorization enabled us to explore potential differences in responses among groups with varying levels of adversity. Similarly, responses to each question on the follow-up questionnaire were analyzed on a continuous scale of 1-7, and consolidated into categories of “agree” or “disagree” with neutral responses excluded. This was done to simplify interpretation and align with the study's objective of assessing general trends in patient agreement or disagreement with key statements. Boxplots were used to visualize the relationship between ACE-Q scores and responses to the follow-up questionnaire. These plots illustrate the distribution of responses, including median values, interquartile ranges, and potential outliers. By capturing variability in participant experiences, boxplots help identify patterns, such as whether individuals with higher ACE-Q scores report increased discomfort or a greater interest in learning about ACEs. This method provides a more nuanced understanding of the data compared to summary statistics alone.

Descriptive statistics demonstrated the distribution of answers to each question, and the distribution of ACE scores across the database. Spearman's rank correlation coefficient was chosen to explore potential relationships between continuous ACE scores and responses to follow-up questions, as it is well-suited for ordinal data. The Kruskal-Wallis H test was used to determine associations between the categorized ACE scores and binary agree/disagree questionnaire answers, as it allows for comparison of non-parametric data across multiple groups.

## Results

### Demographics of Participants

A total of 533 participants completed the ACE-Q only, while 260 participants completed both the ACE-Q and the follow-up questionnaire. Therefore, our response rate was 49%; fairly typical for this study design in primary care.^17^ Of the 260 complete respondents, 75% self-reported being Caucasian, and 84% self-reported being female.

### ACE Scores

**Figure 1.**
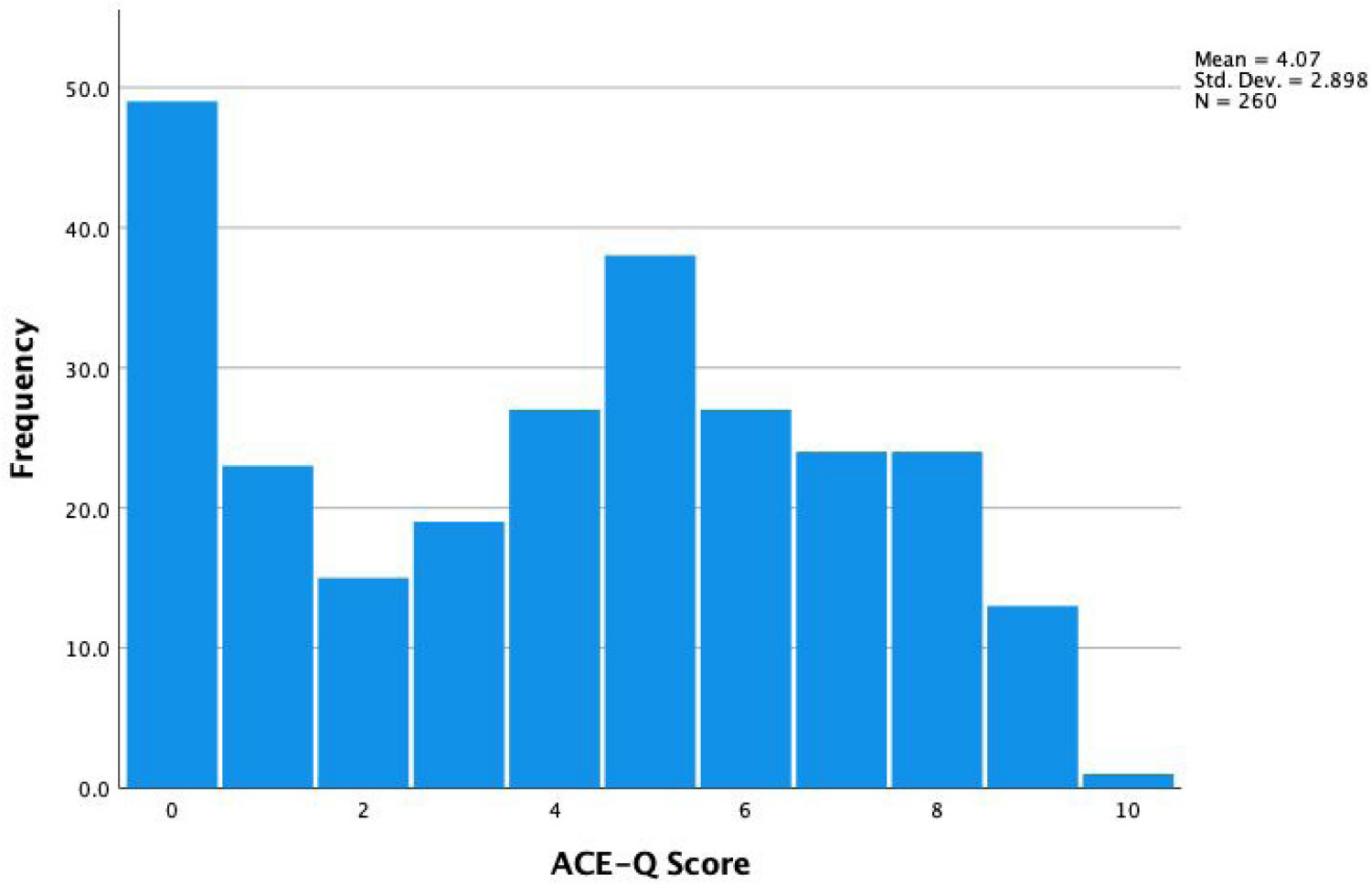
Distribution of ACE-Q scores. ACE-Q, Adverse Childhood Experiences Questionnaire.

Approximately 81% reported an ACE score of at least one, 59% reported an ACE score of four or more (high risk), and 24% reported a score of seven or more (very high risk). Figure 1 shows the distribution of ACE scores across respondents.

Question 1: “I am comfortable being asked about stressful childhood experiences by my health care provider.”

The overwhelming majority of respondents (81.6%) indicated agreement with this statement, and ∼7% indicated a neutral response. There was an indication on Spearman's rank correlation coefficient (*r_s_* = −0.166, *P* = 0.007) that those with higher ACE scores were more likely to disagree with question one.

**Figure 2.**
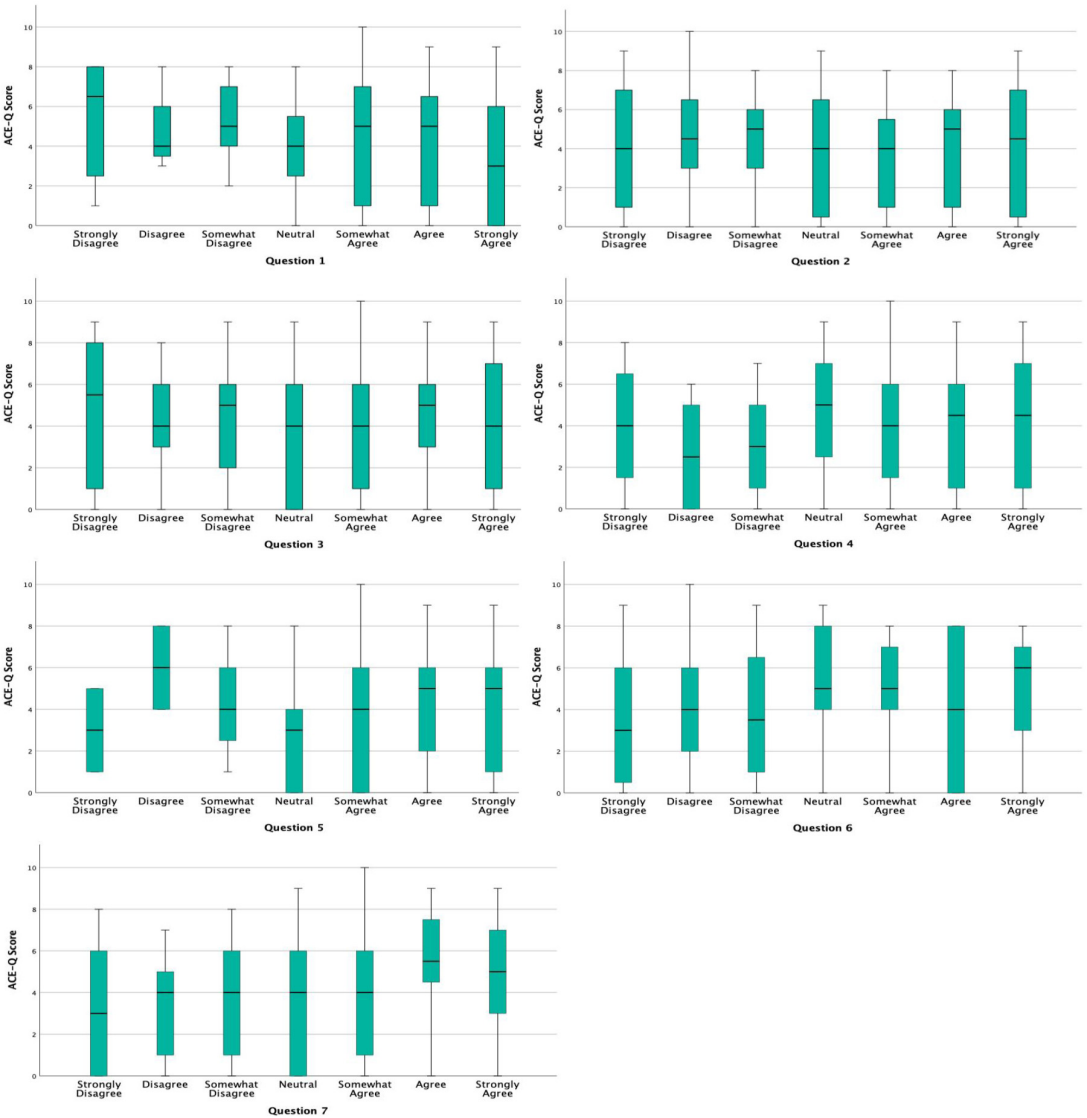
Boxplots of the relationship between ACE-Q score and responses to questions one through seven on the follow-up questionnaire. ACE-Q, Adverse Childhood Experiences Questionnaire.

However, they remained a minority; out of 62 patients with a very high ACE score (7-10), only 10 patients (16%) indicated disagreement (Table 1). Out of 106 patients identified as low risk on ACE screening (0-3), 6 patients (5.6%) indicated disagreement. Figure 2 indicates the distribution of answers to each follow-up question grouped by ACE score. The questions included the follow-up questionnaire can be found in Appendix 2.

Questions 2 and 3: “I would prefer to answer questions about stressful childhood experiences by completing a form without my health care provider in the room.” and “I would prefer that my health care provider ask me face to face about stressful childhood experiences.”

**Table 1.** Frequency of Follow-up Questionnaire Answers for Question One Categorized by ACE-Q Score.

	ACE-Q score	0	1	2	3	4	5	6	7	8	9	10
Question 1, *n*	Strongly Disagree	0	2	0	0	1	0	1	1	3	0	0
	Disagree	0	0	0	1	2	0	0	0	1	0	0
	Somewhat Disagree	0	0	2	1	2	6	1	2	3	0	0
	Neutral	3	0	2	2	3	4	2	0	3	0	0
	Somewhat Agree	13	6	6	4	6	12	6	7	4	7	1
	Agree	11	5	2	4	5	8	9	6	6	3	0
	Strongly Agree	22	10	3	7	8	8	8	8	4	3	0
	Total	49	23	15	19	27	38	27	24	24	13	1

ACE-Q, Adverse Childhood Experiences Questionnaire.

Just over one quarter (∼27%) of individuals reported neutral feelings to both questions. One third of individuals disagreed that they would prefer answering these questions without their provider present, while 22% disagreed that they would prefer answering these questions face-to-face. Conversely, 40% agreed that they would prefer to answer without their provider present, while 50% agreed that they would rather speak with their provider face-to-face. The correlations between ACE score and response for questions two and three were not statistically significant (*r_s_* = −0.039, *P* = 0.534; *r_s_* = −0.007, *P* = 0.915 respectively).

Question 4: “I am comfortable having the results of my ACE screening questionnaire in my chart.”

Most individuals agreed that they would be comfortable having their ACE score as part of their medical record, with >70% expressing agreement, 15% neutral, and only 11% disagreeing. Of those that disagreed, half felt strongly about this. The correlation between ACE score and response for question four was not statistically significant (*r_s_* = 0.20, *P *= 0.751).

Question 5: “I understand why screening for ACEs is important.”

A brief introduction to the screening tool was provided at the beginning of the ACE-Q. We felt it was important to know if those completing the survey had basic knowledge of why ACE scores were important. Reassuringly, greater than 90% of respondents expressed that they understood the importance. The correlation between ACE score and questionnaire response was not statistically significant for this question (*r_s_* = 0.068, *P* = 0.273).

Question 6: “Completing the ACE screening questionnaire made me upset.”

It was important to us to understand whether screening for ACEs was likely to upset a significant portion of those screened. Reassuringly, over 85% either disagreed with, or expressed a neutral response to the statement. Of the 30 individuals that did note discomfort, 80% responded “somewhat agree,” with only 20% responding either “agree” or “strongly agree.” Notably, only 6 out of 260 respondents (2.3%) agreed or strongly agreed that completing the ACE-Q made them upset, indicating that the vast majority did not find the screening distressing. When examining the breakdown of responses by ACE score (Table 2), we saw a strong correlation between high ACE scores and agreement with question six (*r_s_* = 0.173, *P* = 0.005).

Question 7: “I would like more information about ACEs.”

**Table 2.** Frequency of Questionnaire Answers for Question Six Categorized by ACE-Q Score.

	ACE-Q score	0	1	2	3	4	5	6	7	8	9	10
Question 6, *n*	Strongly Disagree	20	11	4	5	7	8	9	7	2	6	0
	Disagree	11	3	7	8	7	8	8	4	6	2	1
	Somewhat Disagree	8	6	3	5	2	8	1	6	2	3	0
	Neutral	5	3	1	1	5	9	5	2	9	2	0
	Somewhat Agree	3	0	0	0	6	5	2	5	3	0	0
	Agree	1	0	0	0	0	0	0	0	1	0	0
	Strongly Agree	1	0	0	0	0	0	2	0	1	0	0
	Total	49	23	15	19	27	38	27	24	24	13	1

ACE-Q, Adverse Childhood Experiences Questionnaire.

Many individuals wanted more information, and agreement on this question was also highly correlated with having a high ACE score (*r_s_* = 0.177, *P* = 0.004).

After categorizing the ACE scores into “low,” “high,” and “very high” and consolidating questionnaire responses into binary “agree” and “disagree” format, there were no statistically significant interactions between ACE category and questions one, two, three, four, five, and seven on the Kruskal-Wallis H test. Question six did show a statistically significant association between higher ACE category and agreement; *H*(2) = 10.526, *P* = 0.005.

## Discussion

ACE scores help assess how early life experiences contribute to long-term disease risk. Despite strong evidence linking ACEs to adverse health outcomes,^4,7^ primary care assessments rarely incorporate childhood trauma as a risk factor. Given that ACEs have been linked to increased risks of chronic disease, mental health conditions, and health care utilization,^1,2,11,13^ routine screening in primary care may enhance early intervention efforts.

One common concern about ACE screening is the potential for re-traumatization or distress.^3,9,18^ However, this concern mirrors early misconceptions in suicide prevention, where direct questioning was once believed to increase risk. Research has since disproven this, showing that asking about suicide directly can aid prevention efforts.^19^ Similarly, structured ACE screening may provide an opportunity to support at-risk individuals rather than cause harm.

While in-depth discussions about past traumatic experiences should generally be reserved for a therapeutic setting with the intent of processing trauma, our team proposes that simply asking whether someone has experienced traumatic events can be beneficial. Screening can help open a dialogue between patients and providers, allowing for a more comprehensive assessment of individual health risks and potential interventions.

Our findings suggest that most primary care patients do not experience significant distress from ACE screening, which aligns with prior research indicating that structured screening in primary care is well tolerated.^11,20^ While those with higher ACE scores were more likely to report discomfort, they also expressed greater interest in learning about ACEs. Importantly, some individuals with high ACE scores reported no distress, while some with low ACE scores found the screening upsetting.

Participants also expressed diverse screening preferences. Some preferred completing the ACE-Q privately, while others favored discussing it face-to-face with their provider. This aligns with best practices in trauma-informed care, which emphasize patient choice and flexibility in screening approaches.^21^ Offering flexible screening options—such as self-administered questionnaires and direct conversations—may improve patient comfort and engagement. Additionally, electronic screening methods can help reduce the risk of well-meaning but uninformed providers probing into specific ACE details, which could inadvertently cause distress.^3^

To improve ACE screening in primary care, provider education is essential. Clinicians must understand the difference between asking about ACE exposure for risk assessment versus engaging in trauma processing, which requires a therapeutic setting.^1,21^ Clarifying this distinction can help ensure that screening remains a tool for identifying health risks rather than a source of re-traumatization. Expanding trauma-informed training programs and developing clear guidelines on how to discuss ACEs with patients could further improve the effectiveness and ethical implementation of screening practices in primary care.

### Limitations

There are several limitations to a survey study of this nature that should be considered when interpreting data. First, due to the nature of our version of the ACE-Q, we are only able to take into consideration the number of ACEs a participant has, not the specific ACEs that one has experienced. Second, our response rate of 49% introduces potential nonresponse bias. Although this rate is typical for primary care survey studies,^17^ it may affect the representativeness of our findings. Additionally, our sample was predominantly composed of white females, which limits the generalizability of our findings to more diverse populations. Research suggests that ACE prevalence and its health impacts may vary by race, ethnicity, and gender,^3^ underscoring the need for broader representation. To address this limitation, future studies should incorporate targeted recruitment strategies, such as community-based outreach and partnerships with clinics serving more diverse patient populations. Finally, this study was conducted at a single-center primary care center, which may limit its applicability to other healthcare settings. Expanding this research to multiple centers across different geographic regions would enhance the generalizability of findings and provide a more comprehensive understanding of ACE screening outcomes.

## Conclusion

Our findings suggest that ACE screening is generally acceptable in a primary care setting, with minimal distress reported by participants. This study provides valuable preliminary insights into patient experiences, highlighting the feasibility of incorporating ACE screening into routine care. While this study does not establish the direct benefits of widespread ACE screening, it underscores its potential to facilitate patient-provider discussions and support trauma-informed care practices. Future multi-center studies are needed to further explore the long-term impact and generalizability of these findings. Nonetheless, this research contributes to a growing body of evidence supporting the integration of ACE screening in primary care and offers guidance for optimizing its implementation.

## Appendix 1: ACE Questionnaire (ACE-Q)

**Adverse Childhood Experiences Questionnaire (ACE-Q)**

Many people experience stressful life events before the age of 18 that increase their risk of physical and mental health concerns as adults. Knowing your ACEs score and understanding your risk can help you and your doctor make the best decisions for your health.

Please read the statements below. Count the number of statements that apply to you and write the total number in the box provided.

Of the statements below, HOW MANY apply to you? Select the total number.

**At any point from birth to age 18…**

- Your parents or guardians were separated or divorced
- You lived with a household member who served time in jail or prison
- You lived with a household member who was depressed, mentally ill, or attempted suicide
- Someone touched your private parts or asked you to touch their private parts in a sexual way that was unwanted, against your will, or made you feel uncomfortable
- More than once, you went without food, clothing, a place to live, or had no one to protect you
- You saw or heard household members hurt or threaten to hurt each other
- A household member swore at, insulted, humiliated, or put you down in a way that scared you OR a household member acted in a way that made you afraid that you might be physically hurt
- You lived with someone who had a problem with drinking or using drugs
- Someone pushed, grabbed, slapped, threw something at you OR you were hit so hard that you were injured or had marks
- You often felt unsupported, unloved and/or unprotected

**TOTAL NUMBER:** [0] [1] [2] [3] [4] [5] [6] [7] [8] [9] [10]

## Appendix 2: ACE Follow-up Questionnaire

Adverse Childhood Experiences (ACEs) are stressful life events occurring before the age of 18 that can cause a dangerous type of stress in the body called “toxic stress,” that increases the risk of developing many different health problems (diabetes, cancer, addiction, depression, etc…) later in life.

Understanding this information can help your doctor better understand your individual risks, and how any symptoms you might have fit into your overall health.

The goal of this questionnaire is to better understand your thoughts and feelings around ACEs, and being asked about them.

**Please indicate how much you agree or disagree with each of the statements below:**

**Table:**

Statement	Strongly agree	Agree	Somewhat agree	Neutral	Somewhat disagree	Disagree	Strongly disagree
I am comfortable being asked about stressful childhood experiences by my health care provider.	☐	☐	☐	☐	☐	☐	☐
I would prefer to answer questions about stressful childhood experiences by completing a form without my health care provider in the room.	☐	☐	☐	☐	☐	☐	☐
I would prefer that my health care provider ask me face to face about stressful childhood experiences.	☐	☐	☐	☐	☐	☐	☐
I am comfortable having the results of my ACE screening questionnaire in my chart.	☐	☐	☐	☐	☐	☐	☐
I understand why screening for ACEs is important.	☐	☐	☐	☐	☐	☐	☐
Completing the ACE screening questionnaire made me upset.	☐	☐	☐	☐	☐	☐	☐
I would like more information about ACEs.	☐	☐	☐	☐	☐	☐	☐

Which culture do you most identify with?

☐ First Nations, Inuit, Métis ☐ Caucasian ☐ Black ☐ South Asian ☐ Asian ☐ Middle Eastern ☐ Hispanic ☐ Other: ___________

Which sex/gender do you identify as?

☐ Male ☐ Female ☐ Other

Thank you very much for taking the time to complete this questionnaire!
