# Corrigendum to “An Assessment of Harm in Adults—Adverse Childhood Experiences Screening in Primary Care: A Survey-Based Study”

**DOI:** 10.1177/23743735251358649

**Published:** 2025-10-07

Inch KM, Olmstead C, Kaschor BA. An Assessment of Harm in Adults—Adverse Childhood Experiences Screening in Primary Care: A Survey-Based Study. *J Patient Exp*. 2025;12. doi:10.1177/23743735251344505

The article has been updated to include the funding information. The updated funding statement is as below:

The authors received no financial support for the research, authorship, and/or publication of this article. This work received funding support for publication from the Office of the Vice Dean, Research and Innovation, Schulich School of Medicine & Dentistry, Western University.

